# Liver Disease Is a Risk Factor for Recurrent Hyperkalemia: A Retrospective Cohort Study

**DOI:** 10.3390/jcm12144562

**Published:** 2023-07-08

**Authors:** Rebecca S. Ahdoot, Jui-Ting Hsiung, Abiy Agiro, Yasmin G. Brahmbhatt, Kerry Cooper, Souhiela Fawaz, Laura Westfall, Kamyar Kalantar-Zadeh, Elani Streja

**Affiliations:** 1 Division of Nephrology, Hypertension, and Kidney Transplantation, Department of Medicine, University of California Irvine, Irvine, CA 92868, USA; ahdoot@hs.uci.edu (R.S.A.); juithsiung@gmail.com (J.-T.H.); 2 Veterans Affairs Long Beach Healthcare System, 5901 East 7th Street, Long Beach, CA 90822, USA; kkalantar@lundquist.org; 3 AstraZeneca, US Evidence, US Medical Affairs, Wilmington, DE 19803, USA; abiy.agiro@astrazeneca.com; 4 AstraZeneca, US Renal, US Medical Affairs, Wilmington, DE 19803, USA; yazzyb342@gmail.com (Y.G.B.); kerry.cooper65@yahoo.com (K.C.); souhiela.fawaz@astrazeneca.com (S.F.); laura.westfall1@astrazeneca.com (L.W.); 5 Harbor UCLA Medical Center, Nephrology, University of California Los Angeles, Los Angeles, CA 90502, USA

**Keywords:** cirrhosis, electrolyte imbalance, hyperkalemia, kidney disease, liver disease, potassium, retrospective study, recurrence

## Abstract

Liver disease is often associated with dysfunctional potassium homeostasis but is not a well-established risk factor for hyperkalemia. This retrospective cohort study examined the potential relationship between liver disease and recurrent hyperkalemia. Patients with ≥1 serum potassium measurement between January 2004 and December 2018 who experienced hyperkalemia (serum potassium >5.0 mmol/L) were identified from the United States Veterans Affairs database. A competing risk regression model was used to analyze the relationship between patient characteristics and recurrent hyperkalemia. Of 1,493,539 patients with incident hyperkalemia, 71,790 (4.8%) had liver disease (one inpatient or two outpatient records) within 1 year before the index hyperkalemia event. Recurrent hyperkalemia within 1 year after the index event occurred in 234,807 patients (15.7%) overall, 19,518 (27.2%) with liver disease, and 215,289 (15.1%) without liver disease. The risk of recurrent hyperkalemia was significantly increased in patients with liver disease versus those without (subhazard ratio, 1.34; 95% confidence interval, 1.32–1.37; *p* < 0.0001). Aside from vasodilator therapy, the risk of recurrent hyperkalemia was not increased with concomitant medication. In this cohort study, liver disease was an independent risk factor strongly associated with recurrent hyperkalemia within 1 year, independent of concomitant renin–angiotensin–aldosterone system inhibitor or potassium-sparing diuretic use.

## 1. Introduction

Chronic liver disease is associated with a significant burden of disease, with >167,000 incident cases and >90,000 related deaths in the United States (US) in 2017 [1]. Among hospitalized US patients, the number of chronic liver disease-related admissions increased by 20.8% between 2012 and 2016 [2]. The incidence and mortality rates for chronic liver disease increased by 30% and 34%, respectively, between 2007 and 2017 [1].

Patients with advanced cirrhosis often experience dysfunctional potassium (K^+^) homeostasis, including hyperkalemia and hypokalemia [3]. In patients with advanced cirrhosis, the prevalence of hyperkalemia is 12–14%, and serum K^+^ levels correlate with markers of impaired kidney function (i.e., serum sodium, urea, and creatinine levels) [3]. Similarly, in hospitalized patients with acute-on-chronic liver failure, the reported incidence of hyperkalemia is 12%, with the presence of acute kidney injury and higher serum K^+^ levels at admission being independent risk factors for hyperkalemia [4]. In these two patient groups, the proportion of male patients was similar among those with or without hyperkalemia [3,4], indicating that the risk of hyperkalemia does not appear to be influenced by patient sex.

As with liver disease, hyperkalemia is also associated with a significant burden of disease, including an increased risk of severe cardiac arrhythmias and mortality [5,6]. Hyperkalemia has been shown to be an independent predictor of mortality in patients with acute decompensation or acute-on-chronic liver disease [7].

Despite the increasing prevalence of the incident chronic liver disease [1] and the significant prevalence and burden of hyperkalemia in these patients [3], liver disease is not a well-established risk factor for hyperkalemia. The aim of this study was to examine the potential relationship between liver disease and recurrent hyperkalemia in a large cohort of patients who had previously experienced hyperkalemia.

## 2. Materials and Methods

### 2.1. Study Design and Objectives

This study retrospectively identified adults (aged ≥ 18 years) from the US Veterans Affairs (VA) database with ≥1 serum K^+^ measurement of 0.5–8.0 mmol/L between January 2004 and December 2018 who had experienced a hyperkalemia event (defined as serum K^+^ > 5.0 mmol/L). Patients with missing demographic, comorbidity, index estimated glomerular filtration rate (eGFR), or medication data were excluded from the analysis.

The objective of this study was to examine the relationship between liver disease and hyperkalemia recurrence within 1 year after the index hyperkalemia event.

Liver disease was defined as the presence of mild-to-moderate or severe liver disease, according to the International Classification of Diseases, ninth and tenth revision, Clinical Modification codes (ICD-9 CM and ICD-10 CM), using one inpatient or two outpatient records within 1 year prior to the index hyperkalemia event. ICD-9 CM and ICD-10 CM codes were also used to identify comorbidities at baseline.

Hyperkalemia recurrence within 1 year after the index hyperkalemia event was defined as ≥1 subsequent serum K^+^ measurement >5.0 mmol/L (taken ≥7 days later), with ≥1 normal serum K^+^ measurement (≤5.0 mmol/L) between the index hyperkalemia event and the recurrence.

### 2.2. Statistical Analysis

Descriptive statistics were used to assess patient characteristics; no formal statistical hypotheses were tested. Mean ± standard deviation (SD) or median (interquartile range [IQR]) were used to describe continuous variables, and counts and percentages were used for categorical variables.

A multivariate Fine and Gray competing risk regression model was used to analyze the relationship between patient characteristics, including demographics (age, sex, race, and ethnicity), eGFR, comorbidities (diabetes, chronic obstructive pulmonary disease [COPD], cancer, congestive heart failure, peripheral vascular disease, cerebrovascular disease, myocardial infarction, liver disease, dementia, rheumatologic disease, peptic ulcer disease, hemiplegia/paraplegia, or acquired immunodeficiency syndrome [AIDS]/human immunodeficiency virus [HIV] infection), and concomitant medications (renin–angiotensin–aldosterone system inhibitor [RAASi], beta-blocker, calcium channel blocker, thiazide, loop diuretic, alpha-blocker, K^+^-sparing diuretic, or vasodilator use), and hyperkalemia recurrence within 1 year after the index hyperkalemia event, in which the outcome was hyperkalemia recurrence and the competing event was all-cause mortality within 1 year after the index hyperkalemia event. Subhazard ratios (sHRs) and 95% confidence intervals (CIs) were used for regression analysis outcomes. Variables in the multivariate model were evaluated jointly and with adjustments for the other variables included. The variables were selected a priori based on clinically relevant risk factors identified in the literature that were captured in the Veterans Affairs database.

### 2.3. Ethical Considerations

This study was approved by the Institutional Review Board of the Tibor Rubin VA Medical Center of Long Beach, CA, USA. Written consent was waived given the research’s nonintrusive nature, large sample size, and patient anonymity.

## 3. Results

### 3.1. Patients

**Figure 1:**
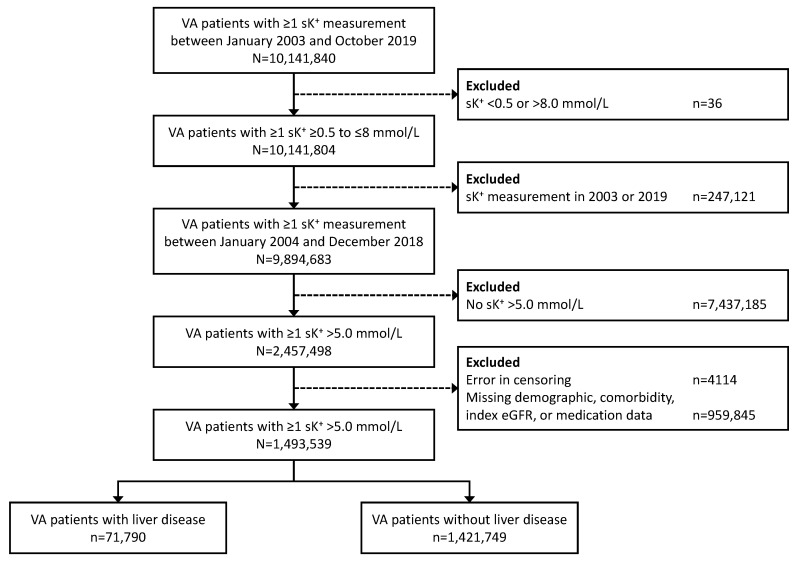
Patient cohort disposition. sK^+^, serum potassium level; VA, Veterans Affairs.

Of 9,894,683 US veterans with ≥1 serum K^+^ measurement of 0.5–8.0 mmol/L between January 2004 and December 2018, we identified 1,493,539 individuals who experienced a hyperkalemia event; of these patients, 71,790 (4.8%) had liver disease prior to the index hyperkalemia event (Figure 1).

The mean ± SD age was 61.5 ± 13.0 years in the total population, 56.4 ± 9.4 years among patients with liver disease, and 61.7 ± 13.1 years in those without liver disease (Table 1). Overall, 96.0% of the patients were male, and the mean ± SD index eGFR was 67.5 ± 25.1 mL/min/1.73 m^2^. Patients with liver disease had a numerically higher median (IQR) Charlson Comorbidity Index than that of those without liver disease (4 (3, 6) vs. 1 (0, 2)). Numerically greater proportions of patients with liver disease also had comorbid diabetes, COPD, cancer, congestive heart failure, myocardial infarction, and AIDS/HIV infection. The use of concomitant medications was similar in patients with and without liver disease.

Patients with liver disease had numerically higher mean ± SD levels of serum alkaline phosphatase (129.8 ± 127.2 vs. 83.5 ± 60.9 U/L), blood urea nitrogen (27.0 ± 21.1 vs. 22.7 ± 15.0 mg/dL), and serum ferritin (587.4 ± 2988.8 vs. 286.1 ± 867.9 ng/mL) than those without liver disease (Table 2). Numerically lower mean ± SD levels of serum bicarbonate (24.9 ± 5.5 vs. 26.9 ± 4.6 mmol/L), total cholesterol (158.5 ± 52.2 vs. 173.8 ± 44.6 mg/dL), low-density lipoprotein cholesterol (89.4 ± 41.9 vs. 99.6 ± 37.9 mg/dL), and triglycerides (143.7 ± 197.8 vs. 150.5 ± 158.8 mg/dL) were observed in patients with versus without liver disease. Other serum electrolyte levels, including serum K^+^, sodium, and calcium, showed non-significant numerical differences between patients with liver disease and those without liver disease.

### 3.2. Hyperkalemia Recurrence

**Figure 2:**
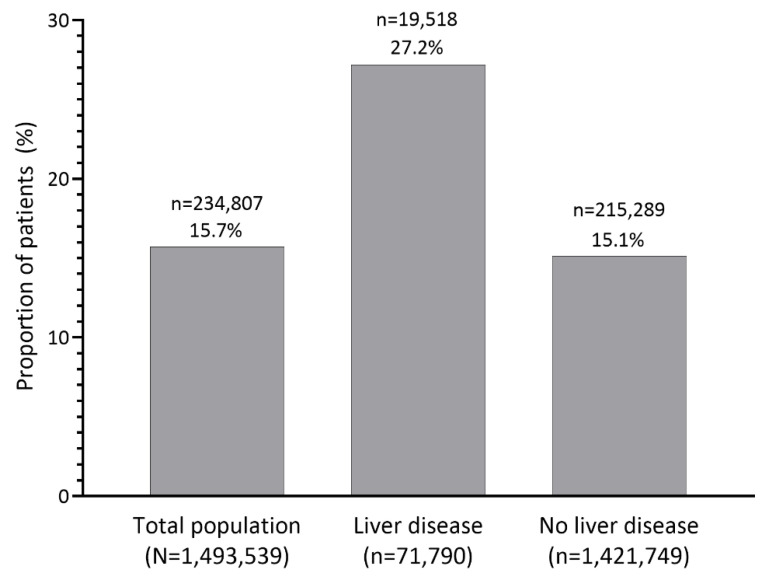
Proportion of patients with recurrent hyperkalemia within 1 year after the index hyperkalemia event in the overall population and in patients with and without liver disease. The number of patients with recurrent hyperkalemia is indicated above the percentage.

In the total population, recurrent hyperkalemia within 1 year after the index hyperkalemia event occurred in 234,807 (15.7%) patients (Figure 2). Recurrent hyperkalemia occurred in 19,518 (27.2%) patients with liver disease and 215,289 (15.1%) patients without liver disease.

### 3.3. Characteristics Associated with Hyperkalemia Recurrence

**Figure 3:**
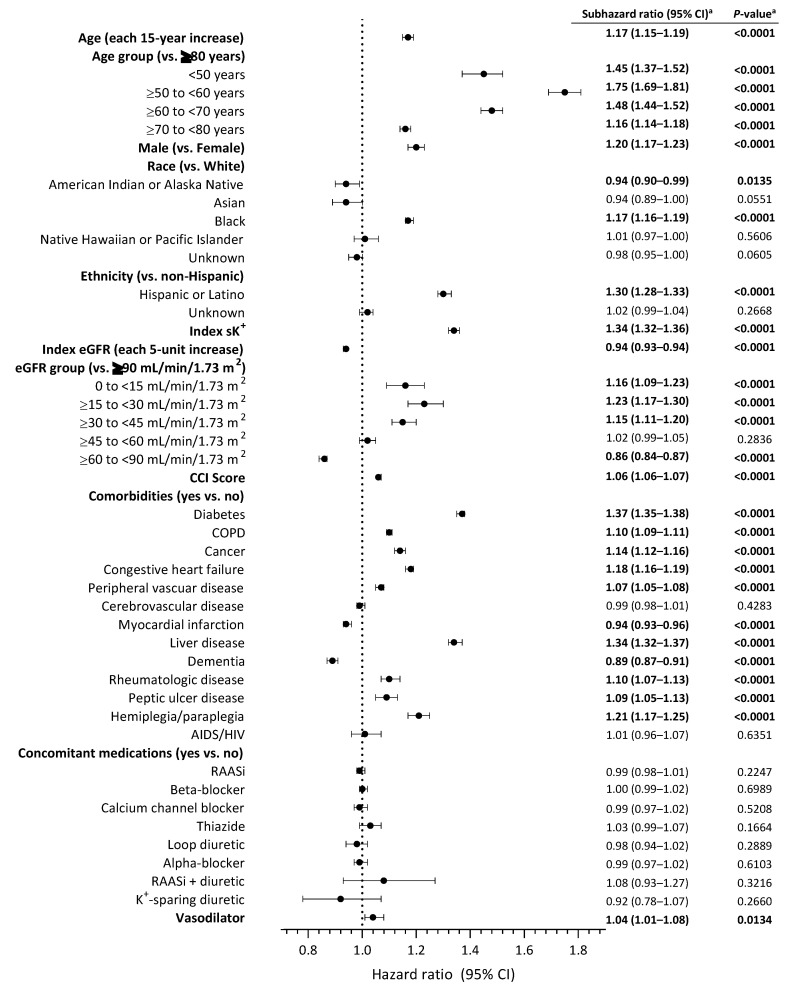
Regression analysis of patient demographic and clinical characteristics and concomitant medications and risk of recurrent hyperkalemia within 1 year after the index hyperkalemia event. ^a^ Statistically significant values are shown in bold text. AIDS, acquired immunodeficiency syndrome; CCI, Charlson Comorbidity Index; CI, confidence interval; COPD, chronic obstructive pulmonary disease; eGFR, estimated glomerular filtration rate; HIV, human immunodeficiency virus; K^+^, potassium; RAASi, renin-angiotensin-aldosterone system inhibitor; sK^+^, serum potassium level.

In the fully adjusted, multivariate, Fine and Gray competing regression analysis, several factors showed a higher risk of recurrent hyperkalemia within 1 year after the index hyperkalemia event compared with their respective referents. The presence of liver disease was associated with a 34% higher risk of the outcome versus the absence of liver disease (sHR, 1.34; 95% CI, 1.32–1.37; *p* < 0.0001) (Figure 3). In this analysis, liver disease had the second strongest association (based on effect estimates) between comorbidity and recurrent hyperkalemia after diabetes (sHR, 1.37; 95% CI, 1.35–1.38; *p* < 0.0001). Other comorbidities were also associated with a higher risk of hyperkalemia recurrence, including hemiplegia/paraplegia, congestive heart failure, cancer, COPD, rheumatologic disease, peptic ulcer disease, and peripheral vascular disease. A lower eGFR category was also associated with recurrent hyperkalemia, with patients in the eGFR 0 to <15, ≥15 to <30, and ≥30 to <45 mL/min/1.73 m^2^ groups having a significantly higher risk of hyperkalemia recurrence than those in the eGFR ≥90 mL/min/1.73 m^2^ group. A higher Charlson Comorbidity Index was also associated with a significantly higher risk of hyperkalemia recurrence (sHR, 1.06; 95% CI, 1.06–1.07; *p* < 0.0001) (Figure 3).

The risk of recurrent hyperkalemia within 1 year of the index hyperkalemia event was significantly increased with concomitant vasodilator therapy (sHR, 1.04; 95% CI, 1.01–1.08; *p* = 0.0134). The risk of recurrent hyperkalemia was not significantly increased with any other concomitant medication in this analysis, including RAASis or K^+^-sparing diuretics. Other significant risk factors for recurrent hyperkalemia were advancing age, younger age group (vs. ≥80 years), male sex (vs. female), black race (vs. white), Hispanic or Latino ethnicity (vs. non-Hispanic), and index serum K^+^ level.

## 4. Discussion

In this large retrospective cohort study of approximately 1.5 million individuals with incident hyperkalemia, the prevalence of recurrent hyperkalemia within 1 year among patients with liver disease was 27.2%, and liver disease was strongly associated with hyperkalemia recurrence, independent of concomitant RAASi and K^+^-sparing diuretic use. Other significant clinical risk factors for hyperkalemia recurrence included diabetes, increased Charlson Comorbidity Index score, lower eGFR, concomitant vasodilator therapy, index serum K^+^ level, and several patient demographics, including advancing age, male sex, black race, and Hispanic or Latino ethnicity.

The prevalence of recurrent hyperkalemia among patients with liver disease in this study was higher than the prevalence of incident hyperkalemia reported in previous studies of patients with advanced cirrhosis (12–14%) [3] or acute-on-chronic liver failure (12%) [4]. This is likely because many patients who develop hyperkalemia are at risk for a recurrent hyperkalemia event, especially those with chronic kidney disease (CKD), chronic heart failure, or diabetes [8].

A previous study showed that the Model for End-Stage Liver Disease (MELD) score, which is considered to be a marker of the severity of hepatic dysfunction, is a stronger predictor for angiotensin receptor blocker-related hyperkalemia than serum creatinine or eGFR alone, with MELD scores of ≥10 being associated with an increased risk of hyperkalemia [9].

In a study of patients at high risk for hyperkalemia (i.e., patients with incident CKD or chronic heart failure or those initiating RAASi therapy), independent predictors of hyperkalemia recurrence were the severity of the index hyperkalemia event, low eGFR levels, diabetes, and spironolactone use [8]. Similarly, our study showed that comorbid diabetes and reduced eGFR were independently associated with recurrent hyperkalemia; however, K^+^-sparing diuretic use was not associated with an increased risk of hyperkalemia recurrence.

The mechanisms underlying hyperkalemia development in patients with liver disease are not fully understood, but reduced liver uptake of potassium, the presence of sarcopenia, use of diuretics or aldosterone antagonists, and a progressive decline in eGFR are thought to be closely related to the increase in hyperkalemia risk in patients with advanced cirrhosis [7]. The development of hyperkalemia is hypothesized to occur via renin–angiotensin–aldosterone system (RAAS) activation, which typically occurs in conjunction with elevated plasma antidiuretic hormone levels, resulting in water and sodium retention, ascites, and hyponatremia [3]. In patients with acute-on-chronic liver disease, hyperkalemia often develops secondary to reduced kidney function, resulting in impaired urinary K^+^ excretion and increased renal sodium and water absorption [3,7]. Hyperkalemia may also be caused by the use of K^+^-sparing diuretics, such as spironolactone or eplerenone [10], which are recommended as first-line treatment in patients with cirrhosis and grade 2 ascites, either alone or in conjunction with a loop diuretic [11]. However, in this analysis, patients with chronic liver disease were at increased risk of hyperkalemia recurrence regardless of concomitant K^+^-sparing diuretic use. The prevalence of hyperkalemia recurrence in our study was higher among patients with liver disease than that in those without liver disease (27.2% vs. 15.1%) independent of RAASi or K^+^-sparing diuretic use and despite the absence of a major between-group difference in serum sodium levels. This suggests that other mechanisms that are independent of the RAAS may play a role in recurrent hyperkalemia in patients with liver disease.

The limitations of this study include its retrospective observational design, which may have led to additional confounding factors that were not included in the regression analysis. Patients treated with potassium binders and those on dialysis accounted for less than 1% each of the study population and were not excluded from this study; due to the small proportions of these patients, we do not believe these to be strong confounders of the presented results. The use of ICD-9 CM and ICD-10 CM diagnosis codes to identify patients with liver disease is another potential limitation, although this would usually be biased toward the null hypothesis [12]. The study population comprised patients treated through the VA health system. As such, the results are not necessarily generalizable to the general US population because of differences in demographics and clinical characteristics between veterans and non-veterans and differences in practice patterns between VA and non-VA health systems.

## 5. Conclusions

In our large retrospective cohort study of US veterans with hyperkalemia, liver disease was an independent risk factor strongly associated with recurrent hyperkalemia within 1 year, independent of concomitant RAASi or K^+^-sparing diuretic use. Further studies are needed to understand the potential mechanisms underlying the association between liver disease and hyperkalemia recurrence.

## Figures and Tables

**Table 1:** Patient demographics and clinical characteristics.

	Total Population (*n* = 1,493,539)	Liver Disease (*n* = 71,790)	No Liver Disease (*n* = 1,421,749)
Age (years), mean ± SD	61.5 ± 13.0	56.4 ± 9.4	61.7 ± 13.1
Sex, *n* (%)			
Male	1,433,710 (96.0)	69,796 (97.2)	1,363,914 (95.9)
Female	59,829 (4.0)	1994 (2.8)	57,835 (4.1)
Race, *n* (%)			
White	1,184,008 (79.3)	48,647 (67.8)	1,135,361 (79.9)
Black	213,725 (14.3)	18,454 (25.7)	195,271 (13.7)
Asian	7832 (0.5)	248 (0.3)	7584 (0.5)
American Indian or Alaska Native	11,741 (0.8)	726 (1.0)	11,015 (0.8)
Native Hawaiian or other Pacific Islander	13,747 (0.9)	638 (0.9)	13,109 (0.9)
Unknown/declined to answer	62,486 (4.2)	3077 (4.3)	59,409 (4.2)
Ethnicity, *n* (%)			
Hispanic or Latino	77,069 (5.2)	4927 (6.9)	72,142 (5.1)
Not Hispanic or Latino	1,364,656 (91.4)	64,410 (89.7)	1,300,246 (91.5)
Unknown/declined to answer	51,814 (3.5)	2453 (3.4)	49,361 (3.5)
Index eGFR (mL/min/1.73 m^2^), mean ± SD	67.5 ± 25.1	67.7 ± 30.7	67.5 ± 24.8
CCI score, median (IQR)	1 (0, 3)	4 (3, 6)	1 (0, 2)
Comorbidities, *n* (%)			
Diabetes	494,831 (33.1)	27,718 (38.6)	467,113 (32.9)
COPD	253,274 (17.0)	20,321 (28.3)	232,953 (16.4)
Cancer	196,206 (13.1)	15,805 (22.0)	180,401 (12.7)
Congestive heart failure	174,229 (11.7)	13,597 (18.9)	160,632 (11.3)
Peripheral vascular disease	126,609 (8.5)	7403 (10.3)	119,206 (8.4)
Cerebrovascular disease	96,643 (6.5)	5474 (7.6)	91,169 (6.4)
Myocardial infarction	77,030 (5.2)	5639 (7.9)	71,391 (5.0)
Dementia	51,398 (3.4)	2803 (3.9)	48,595 (3.4)
Rheumatologic disease	22,575 (1.5)	1237 (1.7)	21,338 (1.5)
Peptic ulcer disease	17,180 (1.2)	2771 (3.9)	14,409 (1.0)
Hemiplegia/paraplegia	17,040 (1.1)	1453 (2.0)	15,587 (1.1)
AIDS/HIV	8720 (0.6)	2010 (2.8)	6710 (0.5)
Concomitant medication, *n* (%)			
RAASi	719,243 (48.2)	34,612 (48.2)	684,631 (48.2)
Beta-blocker	576,679 (38.6)	27,778 (38.7)	548,901 (38.6)
Calcium channel blocker	325,981 (21.8)	15,880 (22.1)	310,101 (21.8)
Thiazides	280,203 (18.8)	13,644 (19.0)	266,559 (18.7)
Loop diuretic	258,552 (17.3)	12,525 (17.5)	246,027 (17.3)
Alpha-blocker	163,954 (11.0)	7969 (11.1)	155,985 (11.0)
RAASi + diuretic	96,985 (6.5)	4679 (6.5)	92,306 (6.5)
K^+^-sparing diuretic	96,011 (6.4)	4647 (6.5)	91,364 (6.4)
Vasodilator	49,515 (3.3)	2416 (3.4)	47,099 (3.3)

AIDS, acquired immunodeficiency syndrome; CCI, Charlson Comorbidity Index; COPD, chronic obstructive pulmonary disease; eGFR, estimated glomerular filtration rate; HIV, human immunodeficiency virus; IQR, interquartile range; K^+^, potassium; RAASi, renin–angiotensin–aldosterone system inhibitor; SD, standard deviation.

**Table 2:** Index laboratory measurements.

Laboratory Measurement ^a^	Total Population (*n* = 1,493,539)	Liver Disease (*n* = 71,790)	No Liver Disease (*n* = 1,421,749)
Serum albumin (g/dL)	4.0 ± 4.1	3.4 ± 1.2	4.0 ± 4.2
UACR (mg/g)	111.0 ± 716.5	258.6 ± 2588.6	107.1 ± 590.3
Serum ALP (U/L)	86.0 ± 67.0	129.8 ± 127.2	83.5 ± 60.9
Serum bicarbonate (mmol/L)	26.8 ± 4.7	24.9 ± 5.5	26.9 ± 4.6
BUN (mg/dL)	22.9 ± 15.3	27.0 ± 21.1	22.7 ± 15.0
Serum calcium (mg/dL)	9.4 ± 0.7	9.0 ± 0.9	9.4 ± 0.7
Serum ferritin (ng/mL)	318.7 ± 1283.8	587.4 ± 2988.8	286.1 ± 867.9
Blood glucose (mg/dL)	129.3 ± 76.4	140.3 ± 98.2	128.8 ± 75.1
Blood glucose POC (mg/dL)	170.1 ± 116.4	168.3 ± 113.9	170.4 ± 116.7
Hemoglobin (g/dL)	13.6 ± 2.2	12.4 ± 2.5	13.7 ± 2.1
HbA1c (%)	6.8 ± 2.5	6.8 ± 2.3	6.8 ± 2.5
Serum sodium (mmol/L)	138.7 ± 5.3	136.1 ± 6.8	138.8 ± 5.2
Serum phosphorus (mg/dL)	3.8 ± 1.3	4.0 ± 1.6	3.8 ± 1.3
Serum K^+^ (mmol/L)	5.3 ± 0.3	5.4 ± 0.4	5.3 ± 0.3
WBC count (× 10^9^/L)	8.6 ± 131.1	10.2 ± 112.9	8.5 ± 132.1
Lipid panel (mg/dL)			
Total cholesterol	173.4 ± 44.8	158.5 ± 52.2	173.8 ± 44.6
HDL-cholesterol	45.3 ± 15.4	42.4 ± 18.5	45.4 ± 15.3
LDL-cholesterol	99.4 ± 38.1	89.4 ± 41.9	99.6 ± 37.9
Triglycerides	150.3 ± 160.0	143.7 ± 197.8	150.5 ± 158.8

^a^ Values presented as mean ± standard deviation. ALP, alkaline phosphatase; BUN, blood urea nitrogen; HbA1c, glycated hemoglobin; HDL, high-density lipoprotein; K^+^, potassium; LDL, low-density lipoprotein; POC, point of care; UACR, urine albumin-to-creatinine ratio; WBC, white blood cell.

## Data Availability

Restrictions apply to the availability of data generated or analyzed during this study. The United States Department of Veterans Affairs places legal restrictions on veterans’ healthcare data, which includes both identifying data and sensitive patient information. The corresponding author will, on request, detail the restrictions and any conditions under which access to some data may be provided. Requests for access to data from this study can be directed to the corresponding author, Elani Streja (estreja@hs.uci.edu).
